# Functional investigation of grass carp reovirus nonstructural protein NS80

**DOI:** 10.1186/1743-422X-8-168

**Published:** 2011-04-14

**Authors:** Lin Cai, Xiaoyun Sun, Ling Shao, Qin Fang

**Affiliations:** 1 State Key Laboratory of Virology, Wuhan Institute of Virology, Chinese Academy of Sciences, Wuhan 430071, China

## Abstract

**Background:**

Grass Carp Reovirus (GCRV), a highly virulent agent of aquatic animals, has an eleven segmented dsRNA genome encased in a multilayered capsid shell, which encodes twelve proteins including seven structural proteins (VP1-VP7), and five nonstructural proteins (NS80, NS38, NS31, NS26, and NS16). It has been suggested that the protein NS80 plays an important role in the viral replication cycle that is similar to that of its homologous protein μNS in the genus of *Orthoreovirus*.

**Results:**

As a step to understanding the basis of the part played by NS80 in GCRV replication and particle assembly, we used the yeast two-hybrid (Y2H) system to identify NS80 interactions with proteins NS38, VP4, and VP6 as well as NS80 and NS38 self-interactions, while no interactions appeared in the four protein pairs NS38-VP4, NS38-VP6, VP4-VP4, and VP4-VP6. Bioinformatic analyses of NS80 with its corresponding proteins were performed with all currently available homologous protein sequences in ARVs (avian reoviruses) and MRVs (mammalian reoviruses) to predict further potential functional domains of NS80 that are related to VFLS (viral factory-like structures) formation and other roles in viral replication. Two conserved regions spanning from aa (amino acid) residues of 388 to 433, and 562 to 580 were discovered in this study. The second conserved region with corresponding conserved residues Tyr565, His569, Cys571, Asn573, and Glu576 located between the two coiled-coils regions (aa ~513-550 and aa ~615-690) in carboxyl-proximal terminus were supposed to be essential to form VFLS, so that aa residues ranging from 513 to 742 of NS80 was inferred to be the smallest region that is necessary for forming VFLS. The function of the first conserved region including Ala395, Gly419, Asp421, Pro422, Leu438, and Leu443 residues is unclear, but one-third of the amino-terminal region might be species specific, dominating interactions with other viral components.

**Conclusions:**

Our results in this study together with those from previous investigations indicate the protein NS80 might play a central role in VFLS formation and viral components recruitment in GCRV particle assembly, similar to the μNS protein in ARVs and MRVs.

## Background

Grass carp reovirus (GCRV) is a tentative member in the *Aquareovirus *genus of the *Reoviridae *family which shares a common genome of 9 to 12 double-stranded RNA (dsRNA) segments packaged within a multilayered icosahedral capsid shell [1]. Different from most identified aquareoviruses, GCRV has been recognized as one of the most pathogenic agents amongst *Aquareovirus *isolates, which has caused a severe epidemic outbreak of hemorrhagic disease affecting a vast majority (~85%) of fingerling and yearling grass carp in southern China [2,3]. GCRV serves as a good model for studying viral replication and pathogenesis of *Aquareovirus *due to its high virulence.

Studies revealed that there is a close evolutionary relationship between MRVs (mammalian reoviruses) and *Aquareovirus *[4]. Recent progress on 3D structural reconstruction of single particles of GCRV by cryo-electron microscopy (CryoEM) confirmed the high similarities in the structures of viral proteins between GCRV and MRVs [5-8]. Previous investigations indicate that GCRV eleven genomic dsRNA segments (named S1-S11) encoded seven structural proteins (VP1-VP7) and five nonstructural proteins (NS80, NS38, NS31, NS26, and NS16) [4]. Comparative studies revealed that mature GCRV particles consists of five core proteins VP1, VP2, VP3, VP4, and VP6 which are respectively homologous to λ2, λ3, λ1, μ2, and σ2 in MRVs; and two outer capsid proteins VP5 and VP7 which are analogues of μ1 and σ3 in MRVs [4-7]. However, there is no special cell attachment protein in GCRV that is homologous to MRVs σ1, hinting at different viral attachment mechanisms in GCRV and MRVs.

While the structural proteins are of great importance for GCRV entry into cells during its infection, nonstructural proteins are believed to be indispensable in replication and nascent virus particle assembly. Recent progress on nonstructural proteins in genus *Orthoreovirus *including MRVs and ARVs (avian reoviruses) suggests that μNS serves as a versatile protein which could form viral factory-like structures (VFLS) and recruit proteins σNS, λ1, λ2, λ3, μ2, σ2, and core particles to VFLS [9-21]. As such, NS80 might act as a scaffolding protein and a leading performer associated with the highly efficient viral replication and/or accurate assembly of progeny core particles during GCRV infection. Apart from the central role played by μNS in VFLS formation, nonstructural protein σNS, core protein μ2 and σ2 as well as RNA transcription related proteins λ1, λ2, and λ3 are also crucial accessories in VFLS formation in associating with μNS[17,20]. σNS is known to interact directly with μNS and act as a plus single-stranded RNA (ssRNA) binding protein involved in recruiting mRNAs to VFLS for reovirus genomic dsRNA synthesis and assembly [10,21-23]. The core protein μ2 is known to be a cellular microtubule associated protein that is linked to the VFLS morphology formation (showed either globular or filamentous). It displays both ssRNA and dsRNA binding abilities that might function in reovirus transcription [11,24-30] and also exhibits both nucleoside and RNA triphosphatase activities[28,29]. Moreover, another core protein σ2 that is known to play a role in stabilizing the core shell and outer capsid, has also been detected to interact with μNS and performs dsRNA-binding ability but shows no enzymatic activity [21,31,32].

Much progress has been made in identifying the multiple roles played by μNS in viral replication and assembly in MRVs/ARVs, but little is known about the properties of NS80 protein in *Aquareovirus*. Previously, we characterized that the full-length and carboxyl-proximal region (aa 335-742) of NS80 are able to form VFLS in both infected and transfected cells[33]. To extend our understanding of this multifunctional protein, we investigated interactions of NS80 with itself and with the proteins NS38, VP4 and VP6 using the yeast two-hybrid system. Bioinformatic analysis was also conducted to identfy and define the potential functional domains of NS80 that are related to VFLS formation and other roles in GCRV replication and assembly. Our results provide valuable information for further detailed experimental design of NS80 to determine the functional domains and explore currently unknown functions.

## Methods

### Virus and cell lines

GCRV strain named GCRV873, maintained in this laboratory was used in this study. CIK (*Ctenopharyngodon idellus kidney*) cells were employed for GCRV propagation. The GCRV particles were purified from the infected CIK cells and used for genomic RNA extraction. The first strand cDNA was synthesized using GCRV total genomic RNA as a template that is described elsewhere [3].

### Construction of recombinant plasmids

The prepared total cDNA of GCRV873 was used as the PCR (Polymerase Chain Reaction) template. A total of four pairs of primers (listed in Table 1) were designed and used in the PCR amplification of GCRV873 target genes encoding proteins of NS80 (Y2H-NS80-F and Y2H-NS80-R), NS38 (Y2H-NS38-F and Y2H-NS38-R), VP4 (Y2H-VP4-F and Y2H-VP4-R), and VP6 (Y2H-VP6-F and Y2H-VP6-R). The PCR amplification was performed using an Eppendorf Mastercycler personal (Hamburg, Germany) in a 50 μL volume containing ~10 ng cDNA, 1 ng μL^-1 ^of each primer, 200 μM of each dNTP, 10 μL of 5 × PrimeSTAR™ Buffer and 1.25 units of PrimeSTAR™ HS DNA Polymerase (TaKaRa, Dalian, China), and ddH_2_O up to 50 μL. The PCR program consisted of an initial 2 min denaturation step at 98°C; 30 cycles of 10 sec at 98°C, 2 min at 68°C; and a final extension step at 68°C for 5 min. The positive gene fragments were purified by AxyPrep™ DNA Gel Extraction Kit (Axygen, Hangzhou, China). Clontech Y2H bait plasmid pGBKT7, prey plasmid pGADT7, gel purified gene fragments encoding NS80 and NS38 were double digested at 37°C for 3 hours in a 50 μL volume containing 5 μL of 10 × buffer, 1 μL of *Eco*RI (TaKaRa, Dalian, China), 1 μL of *Bam*HI (TaKaRa, Dalian, China), 2-5 μg of the vector or gene fragment, amending ddH_2_O to 50 μL. The pGBKT7 and pGADT7 vectors, gel purified gene fragments encoding VP4 and VP6 were double digested with *Nde*I (TaKaRa, Dalian, China) and *Sma*I (TaKaRa, Dalian, China) using the same volume and condition as the above. The digested products were heated at 70°C for 10 min and then purified using AxyPrep™ PCR Cleanup Kit (Axygen, Hangzhou, China). Here, it is important to note that *Eco*RI and *Nde*I sites have the same fusion frame in both the pGBKT7 and pGADT7 vectors. After estimating the concentrations by running an agarose gel of the digested and purified vectors and inserted fragments of interest, ligations were performed and incubated at 4°C overnight and transformed into *E.coli *DH5α competent cells by the chemical transformation method according to standard protocol. The transformants were screened by using regular enzyme digestion and PCR methods, and the resulting candidate positive clones were confirmed using ABI 3730XL DNA sequencer by Invitrogen Biotechnology Co., Ltd (Shanghai, China).

**Table 1:** Primers used for PCR amplification and sequencing of GCRV target gene in this study

Primer	Sequence	Restriction enzyme
For cloning		
Y2H-NS80-F	5'-AAAGAATTCATGGCACGCCGCATTACT-3'	*Eco*RI
Y2H-NS80-R	5'-AAAGGATCCTTACAGCAGCAGGGAGGC-3'	*Bam*HI
Y2H-NS38-F	5'-AAAGAATTCGCTTACCGATTGACAACC-3'	*Eco*RI
Y2H-NS38-R	5'-AAAGGATCCACACCCTTACATACCC-3'	*Bam*HI
Y2H-VP4-F	5'-AAACATATGATCACCATTGTGGTTATT-3'	*Nde*I
Y2H-VP4-R	5'-AAACCCGGGCTCAAACCCCGGTCGAG-3'	*Sma*I
Y2H-VP6-F	5'-AAACATATGGCACAGCGTCAGTTTTTCG-3'	*Nde*I
Y2H-VP6-R	5'-AAACCCGGGTTAGACGAACATCGCCTG-3'	*Sma*I
For sequencing		
T7	5'-TAATACGACTCACTATAGGGC-3'	
3' DNA-BD	5'-ATCATAAATCATAAGAAATTCGCC-3'	
3' AD	5'-AGATGGTGCACGATGCACAG-3'	
NS80-walking	5'-ATCTCGCTCCAGCACCCT-3'	
VP4-walking	5'-ACTACTACCTCCAGTGGC-3'	

F, forward primer; R, reverse primer.

### In vivo yeast two-hybrid assay and identification

Matchmaker™ GAL4 Two-Hybrid System 3 Kit, yeast selective media and other necessary reagents used in yeast transformation and Y2H assay were purchased from Clontech Laboratories, Inc. After testing the bait and prey constructs for autoactivation and toxicity, a total of twelve yeast co-transformations were performed to identify interactions of NS80-NS80 (pGBKT7-NS80 & pGADT7-NS80), NS80-NS38 (pGBKT7-NS80 & pGADT7-NS38), NS80-VP4 (pGBKT7-NS80 & pGADT7-VP4), NS80-VP6 (pGBKT7-NS80 & pGADT7-VP6), NS38-NS38 (pGBKT7-NS38 & pGADT7-NS38), NS38-VP4 (pGBKT7-NS38 & pGADT7-VP4), NS38-VP6 (pGBKT7-NS38 & pGADT7-VP6), VP4-VP4 (pGBKT7-VP4 & pGADT7-VP4), VP4-VP6 (pGBKT7-VP4& pGADT7-VP6), VP6-VP6 (pGBKT7-VP6& pGADT7-VP6), 53-T (pGBKT7-53 & pGADT7-T, positive control), and Lam-T (pGBKT7-Lam & pGADT7-T, negative control). Briefly, for each transformation, 100 ng of bait recombinant plasmid and 100 ng of prey recombinant plasmid were mixed and transformed into *Saccharomyces cerevisiae *AH109 in small scale reactions according to the Clontech Kit User Manual (PT3247-1). The transformants were then grown at 30°C on SD (synthetically defined) medium. Each yeast transformation was grown on both plates of SD/-Leu-Trp (Double Dropout, ab. DDO) and SD/-Ade-His-Leu-Trp (Quadruple Dropout, ab. QDO).

Yeast AH109 transformants were inoculated into 5 mL DDO broth and incubated, shaking at 30°C until OD_600 _reached an approximate concentration of 0.4-0.6. Aspirated 5 μL of the each cultured transformant and inoculated onto both plates of DDO and QDO containing 1 × BU salts (pH 7.0) and X-Gal (ab. DDO/X and QDO/X, respectively). The remaining cultures were centrifuged and used to isolate the co-transformed plasmids by E.Z.N.A.™ Yeast Plasmid Kit (Omega, USA) and PCR identification performed. For preparation of yeast cultures and protein extracts, we referred to the Clontech Yeast Protocols Handbook (PT3024-1). The Urea/SDS method recommended in this instruction was used to extract yeast total proteins. For Western blotting, NS80 specific antiserum that was previously described was employed as primary antibody [33]. The alkaline phosphatase coupled goat anti-rabbit IgG (Sigma, USA) was used as secondary antibody. The result was visualized by developing with NBT/BCIP AP substrate solution (Promega, USA) according to the manufacturer's instructions.

### Phylogenetic and bioinformatic analyses

For phylogenetic analysis, NS80 deduced amino acid sequence and related reference sequences were checked manually and edited by using ClustalX software [34].The MEGA 3.1 software package was used to construct NS80's phylogenetic tree by the neighbor-joining distance method [35]. The reliability of the inferred tree was tested with 1000 bootstrap replicates.

For bioinformatic analyses, a total of three online programs were used to analyze sequences of NS80 and its homologues extracted from NCBI GenBank database. NS80 primary structure alignment was performed by running the NCBI blastp search tool (http://blast.ncbi.nlm.nih.gov/Blast.cgi). The Multicoil program was used to predict NS80 α-helical coiled-coil regions and generate custom figures (http://groups.csail.mit.edu/cb/multicoil/cgi-bin/multicoil.cgi). Multiple sequence alignment (MSA) was performed by the ClustalW program to obtain conserved regions and residues (http://myhits.isb-sib.ch/cgi-bin/clustalw).

## Results

### Identification of GCRV NS80, NS38, VP4, and VP6 interactions by Y2H assay

GCRV NS80, NS38, VP4, and VP6 encoding genes were cloned into the Clontech Y2H bait plasmid pGBKT7 and prey plasmid pGADT7 respectively, and the following eight constructs: pGBKT7-NS80, pGADT7-NS80, pGBKT7-NS38, pGADT7-NS38, pGBKT7-VP4, pGADT7-VP4, pGBKT7-VP6, and pGADT7-VP6 were successfully obtained. The validities of these recombinant plasmids were confirmed by sequencing.

**Figure 1:**
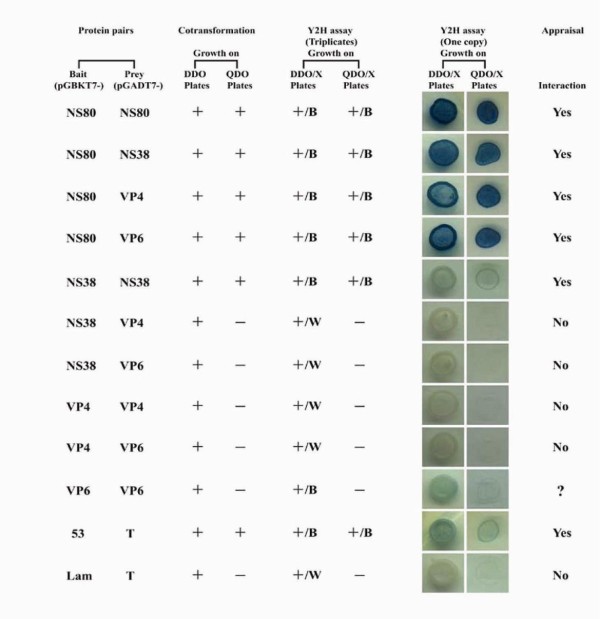
**Yeast co-transformation and Y2H phenotype assessment of NS80, NS38, VP4, and VP6 interactions**. Only yeast transformants that grew on both DDO and QDO plates and formed blue colonies could represent positive interaction. '+ or -' indicates 'growth or ungrowth'. 'B or W' corresponds to 'blue or white'.

For yeast AH109 co-transformation assay, bait-prey protein pairs of NS80-NS80, NS80-NS38, NS80-VP4, NS80-VP6, NS38-NS38, 53-T (positive control) could grow on both DDO and QDO plates, however, NS38-VP4, NS38-VP6, VP4-VP4, VP4-VP6, VP6-VP6, and Lam-T (negative control) could grow on DDO plates but not QDO plates (summarized in Figure 1). Expression of interacting proteins in co-transformed AH109 strain must have the ability to activate all report genes and to be cultured on both DDO and QDO plates. Hence, only protein pairs of NS80-NS80, NS80-NS38, NS80-VP4, NS80-VP6, NS38-NS38, and 53-T could be the candidates of positive interactions. As we know, interacting proteins constructs in yeast transformants could not only grow on both DDO and QDO plates but also form blue colonies. According to the phenotype assessment on both DDO/X and DDO/X plates (Figure 1), the NS80 protein could interact with itself, NS38, VP4, or VP6. The NS38 protein interacted with itself but not with VP4 or VP6. The VP4 protein had no interaction with itself or VP6. We could not make a conclusion for self-interaction of the VP6 protein, as transformants could not grow on QDO plates although they could grow on DDO plates and formed blue colonies.

To analyze yeast plasmid inserts, all the respective co-transformed bait and prey recombinant plasmids were isolated and could be amplified by PCR identification (data not shown). Moreover, the NS80 protein expression could be detected by Western blotting using NS80 rabbit antiserum as the first antibody (data not shown). These results indicated that the bait and prey plasmids could well replicate and express in yeast cells.

### Phylogenetic characterization of GCRV NS80

**Figure 2:**
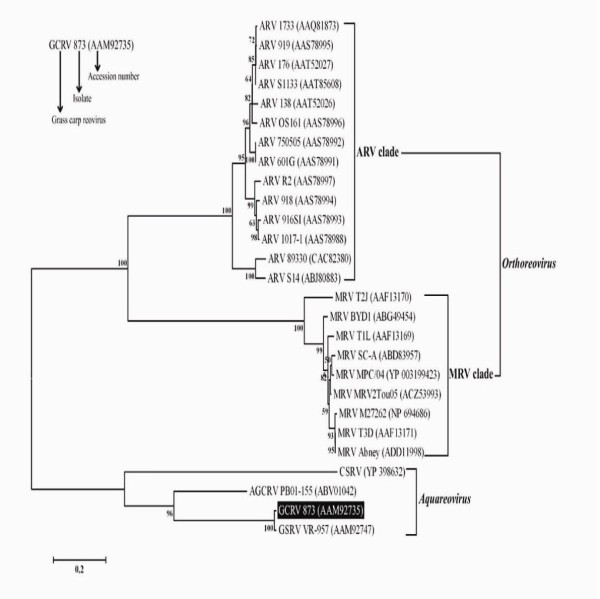
**Phylogenetic analysis based on sequence alignment of GCRV NS80 and its homologues**. NS80 is highlighted in black and bootstrap values over 50% are shown. The scale bar 0.2 means 20% amino acids sequence substitution.

In order to understand the evolutionary relationship between the NS80 protein and other known homologues, phylogenetic analysis was performed. A representative phylogenetic tree based on deduced amino acid sequences of NS80 and its homologues was constructed (Figure 2). The tree was divided into three distinct clades: the ARV clade, the MRV clade, and the *Aquareovirus *clade. The ARV clade and the MRV clade were close members belonging to the *Orthoreovirus *genus, but they were phylogenetically distant from the *Aquareovirus *clade. Phylogenetic analysis of NS80 also revealed that the ARV clade was closer to the *Aquareovirus *clade than to the MRV clade which seemed to be similar to the phylogenesis of host species that belong to Aves, Pisces, and Mammalia, respectively.

### Bioinformatic analyses of GCRV NS80

**Figure 3:**
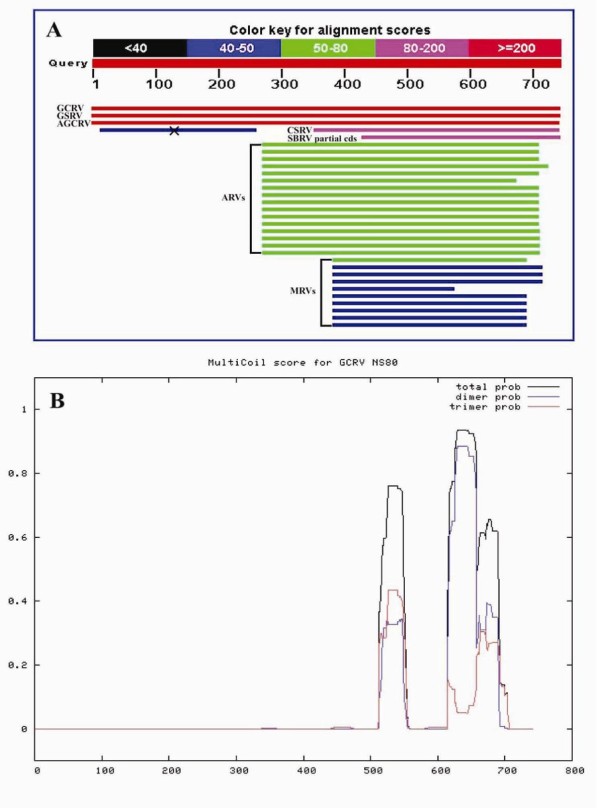
**Alignment of GCRV NS80 with its homologous proteins**. (A) Blastp analysis of GCRV NS80. GSRV (golden shiner reovirus), AGCRV (American grass carp reovirus), CSRV (chum salmon reovirus), SBRV (striped bass reovirus). ' × ' indicates incompatible alignment by accident. 'Red, pink, green, blue, and black' colours represents alignment scores of '≥200, 80-200, 50-80, 40-50, and <40', respectively. (B) Prediction of GCRV NS80 coiled-coil regions by Multicoil program. The x-axis and y-axis displays amino acid position and coiled-coil probabilities, respectively.

**Figure 4:**
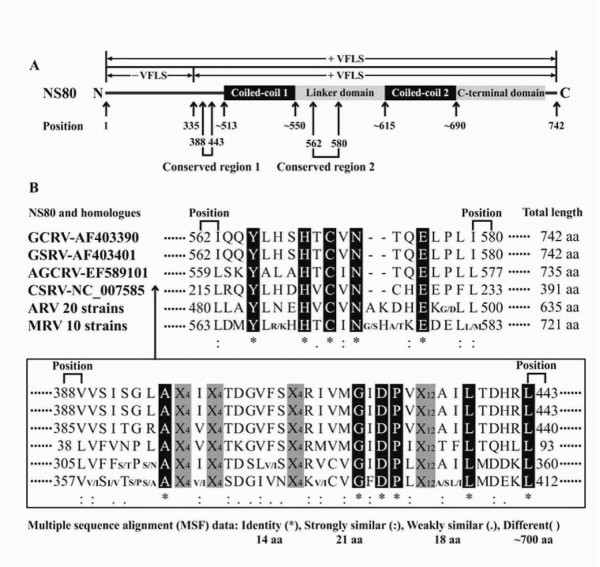
**Schematic representation of GCRV NS80 conserved regions and potential functional domains**. (A) NS80 conserved regions and potential functional domains. '+ (-) VFLS' indicated formation (not formation) of viral factory-like structures. (B) Multiple sequence alignment of NS80 and its homologues. Conserved residues were highlighted in black. 'X_4 _or X_12 _(painted in gray)' respectively showed four or twelve amino acid residues without any similarities.

Bioinformatic analyses were performed to further explore the roles of NS80 in GCRV genome replication and particle assembly. Pairwise comparisons of NS80 with homologues of ARVs or MRVs revealed amino acid identity ranging from 24% to 25%, or 21% to 22%, respectively. Both NCBI blastp (Figure 3A) and ClustalW MSF analysis (Figure 4B) indicated that NS80 and its homologues only had identities in their carboxyl-proximal region which has already been proved sufficiently to form VFLS in MRVs [17,20]. According to the Multicoil prediction program, two α-helical coiled-coils (aa ~513-550 and aa ~615-690, respectively) were also predicted in the carboxyl-proximal terminus region (Figure 3B). Two conserved regions (aa ~388-443 and aa ~562-580, respectively) and corresponding conserved residues (Ala395, Gly419, Asp421, Pro422, Leu438, and Leu443 for the former; Tyr565, His569, Cys571, Asn573 and Glu576 for the latter) were discovered in the NS80 protein by MSA analysis (Figure 4B). So far as we known, the first conserved region and corresponding residues have not been reported to be associated with any known functions of NS80 or its homologues in MRVs and ARVs. However, the second conserved region (located in the linker domain, Figure 4A) and the two predicted coiled-coils have been shown to be required for forming VFLS in both MRVs and ARVs [17,20]. According to the above findings, we conclude that the minimum region of GCRV NS80 required for the VFLS formation should be in the range of amino acid residues from ~513 to 742. The one third of the NS80 amino region with no identities or implications (Figure 3) is thought to interact with other proteins or nascent RNA that is facilitated for VFLS formation.

## Discussion

Since Fields and Song firstly described the yeast two-hybrid system [36], it has been become a powerful genetic approach to identify protein-protein interaction (PPI) *in vivo*. The system can be applied in three scenarios: novel PPI identification, putative PPI confirmation, and definition of interacting domains. For application in reovirus PPI identification, Becker *et al*. reported MRV μNS could interact with σNS but not with outer capsid protein σ3 using the Clontech Y2H system [12]. Another recent associated investigation indicated that ARV μNS could interact with itself using the Clontech mammalian two-hybrid system [20]. In this study, the protein NS80 was first identified to have interactions with NS38, VP4 and VP6 except for NS80 and NS38 self-interactions, indicating that the NS80 and NS38 proteins are capable of both homologous and heterogenous interactions. At the same time, no interactions could be detected between protein pairs NS38-VP4, NS38-VP6, VP4-VP4, VP4-VP6, suggesting dominant protein interactions happened in NS80 during the viral life cycle. However, for VP6 self-interaction, we could not draw a definite conclusion, since three reporter genes including *HIS3 *(synthesis of histidine), *ADE2 *(synthesis of adenine), and *lacZ *(expression of β-galactosidase) should be simultaneously activated to define positive interactions, but only *lacZ *was expressed in the Y2H assay. Taking into consideration that blue colonies formed on DDO/X plates, maybe this was caused by protein misfolding, and further confirmation, by using another PPI system is necessary.

Bioinformatic analysis is a very useful tool for predict protein function and functional domains and was applied to provide information related to NS80 function. Blastp analysis indicated GCRV NS80 and MRVs/ARVs μNS only displayed weak identities in the carboxyl-proximal region, however, this region is required for formation of VFLS of NS80 in *Aquareovirus *and μNS in *Orthoreovirus *and remains relatively conserved amongst different host species. It has been proved that the MRVs μNS carboxyl-terminal one-third region (aa 471-721) is the smallest region necessary for forming VFLS [17], and the GCRV NS80 carboxyl-terminal region (aa 335-742) has been characterized to have the same phenotype[33]. It is important to note, however, this region spanning from aa residues 335 to 742 is apparently sufficient but not essential for this phenotype. Based on our analysis, the necessary or smallest region for VFLS formation in NS80 is located in aa residues 513-742, although this needs to be experimentally confirmed in subsequent studies. We also note that it has been reported that the second conserved region of NS80 and five conserved amino acid residues (Tyr565, His569, Cys571, Asn573 and Glu576) located between the two coiled-coils are thought to be necessary and crucial to the VFLS formation, and which has been proved to cause the loss of the VFLS formation phenotype in MRVs/ARVs by mutating either the conserved His or Cys residue or by truncating this region [17,20].

As previously reported in MRVs, the first 40 residues of the μNS N-terminus are sufficient for interaction with both σNS (NS38's homolog) and μ2 (VP4's homolog), and the region of 173-221 residues is sufficient for interaction with σ2 (VP6's homolog) [21]. Although we have experimentally proved NS80 interacts with NS38, VP4, and VP6, we could not predict the interacting domains because no conserved residues were discovered in the corresponding position by either blastp or MSF analyses. The μNS amino-terminal region of 1-221 residues was sufficient to interact with σNS, λ1, λ2, μ2, and σ2 [21], however, no conserved residues were identified in the NS80 amino-terminal region by aligning analogous proteins from the genera of *Aquareovirus *and *Orthoreovirus *using the ClustalW program, which indicates the amino-terminal region is highly divergent. It is likely that the N-terminal functional regions of NS80 are species special to dominate interactions with related components.

The NS80/μNS is approximately divided into three regions, the carboxyl-terminal one-third region is involved in VFLS formation and the amino-terminal one-third region may be associated with protein recruitments. But the roles of the middle one-third region have never been investigated and remain unknown. Based on our bioinformatic analysis, the presence of a conserved region (aa ~388-443 for NS80) including six conserved amino acid residues (Ala395, Gly419, Asp421, Pro422, Leu438, and Leu443 for NS80) located in this region is reported here for the first time, which suggests novel functions might be explored in NS80/μNS.

In this study, we established that NS80 interacted with itself, NS38, VP4, or VP6 and the NS38 self-interaction, characterized the phylogenesis of NS80, and proposed the potential functional regions. The results contribute a basic understanding of NS80 VFLS formation and interaction with other proteins during GCRV replication and particle assembly, moreover, the study provides valuable information for further experimental design and investigation of NS80 functions.

## Conclusion

Based on our investigation, it is suggested that GCRV NS80 shares common roles with MRVs/ARVs μNS in VFLS formation and other viral components recruitment during viral replication and assembly. Our findings give a better understanding of NS80 functions and also provide guidance for the further study of this multifunctional protein.

## Competing interests

The authors declare that they have no competing interests.

## Authors' contributions

QF and LC designed the experiments. LC carried out the yeast two-hybrid (Y2H) experiment and the bioinformatic analysis. XS and LS participated in partial work of recombinant plasmid construction and Y2H identification. LC and QF analyzed the data and wrote the paper. All authors read and approved the final manuscript.
